# The nitrate content of fresh and cooked vegetables and their health-related risks

**DOI:** 10.1371/journal.pone.0227551

**Published:** 2020-01-09

**Authors:** Hamzeh Salehzadeh, Afshin Maleki, Reza Rezaee, Behzad Shahmoradi, Koen Ponnet

**Affiliations:** 1 Student Research Committee, Kurdistan University of Medical Sciences, Sanandaj, Iran; 2 Environmental Health Research Center, Research Institute for Health Development, Kurdistan University of Medical Sciences, Sanandaj, Iran; 3 Faculty of Social Sciences, imec-mict-Ghent University, Ghent, Belgium; ICAR- Indian Agricultural research Institute, INDIA

## Abstract

**Background:**

Vegetables are the most important source of nitrates in the human diet. During various processes in the body, nitrates are converted into nitrites, which causes various diseases, such as blue baby syndrome and cancer. This study aimed to determine the concentration of nitrates in several vegetable farms in Sanandaj city and to evaluate their health-related risks.

**Methods:**

This descriptive cross-sectional study was conducted from October 2017 to July 2018. A total of 90 samples were taken from nine farms. Soil and water sampling was also carried out. All stages of sample preparation and extraction were carried out according to Food Standards 2–16721, and the nitrate measurements were performed using ion chromatography (Compact IC Plus 882 Model, Metrohm, Switzerland). A health risk assessment was performed using the non-carcinogenic risk assessment.

**Results:**

This study’s results showed that the nitrate concertation in all vegetables was less than National Iranian Vegetable Nitrate Standard. Nitrate levels in leafy vegetables were higher than in root vegetables, and the root vegetables levels were higher than those in Fruit vegetable. The nitrate level in vegetables in autumn was higher than in spring. The cooking process reduced the raw vegetables’ nitrate content from 4.094% to 13.407%, while the frying process increased the vegetables’ nitrate content from 12.46% to 29.93%. The highest health risk level in raw, cooked and fried vegetables was parsley, parsley and beet leaves, respectively, and the lowest in all categories was tomatoes. Generally, the highest health risk was related to fried beet leaves, and the lowest was raw tomatoes. In addition, each of the abovementioned relationships between vegetables’ nitrate levels and the harvest season, type of processing procedure and type of vegetables was significant (*p* < 0.05). The irrigation water’s nitrate concentration in all fields was between 12.36 and 33.14 mg/l. The soil contained nitrate levels of between 4.35 and 9.7 mg/kg.

**Conclusion:**

Based on this study, we can conclude that the amount of nitrates in raw vegetables was lower than the standard limit’s level and that this level does not cause health problems for consumers.

## Background

Vegetables are a rich source of vitamins, minerals and antioxidants. The anticancer properties of vegetables and their ability to reduce cardiovascular disease have been proven [1]. Therefore, ensuring that vegetables are cultivated in a qualitative way is essential for a community’s health. The presence of nitrates in food and their harmful effects on human health is highly debatable [2]. Nitrates exist in a wide range of foods [3]. The nitrate amount in food is quite variable from one region to another and depends on many factors, including the cultivation frequency, the weather conditions, the soil quality, the food production processes and the type and amount of fertilizers used. [4]. Vegetables are the most important source of nitrate exposure in the human diet and contribute to the intake of more than 80% of nitrates [1, 5]. Excessive usage of nitrogenous fertilizers has increased the nitrate concentration in various vegetables. The nitrate concentration in vegetables differ depending on various factors, such as the amount and number of applications of nitrogen-containing fertilizers for soil fertility, growth conditions, weather conditions, season, temperature, light intensity, cultivation type (traditional versus greenhouse), harvesting time, moisture stress, plant species, plant age, soil pH, storage conditions and post-harvest storage [6–8]. So far, many studies have been conducted on the nitrate concentration in vegetables. For example, Ayaz and colleagues demonstrated that the highest nitrate content was found in parsley and spinach, and the lowest nitrate content was found in tomatoes [9]. Another study by Prasad and colleagues showed that the boiling process reduced the nitrate levels in fresh leafy vegetables by 47–59%, but the process of frying in soybean oil increased the nitrate content by 159–309%. Moreover, when the flash -freezing method was used, a slight change was observed in the amount of nitrate in leafy vegetables during a seven-day period [10]. The presence of nitrates in foods has led to various complications, including stomach, intestine, bladder and mouth cancers; fetal birth defects; and methemoglobinemia in children. [11, 12]. Different standards have been proposed for a maximum permitted concentration of nitrates in vegetables, most notably the European Commission Regulation (No. 194/97), which was established in 1997 [13]. There are, however, no standards in Iran yet. The maximum nitrate amount to be ingested daily is less than 3.65 mg/kg by body weight. Accordingly, a person with an average weight of 70 kg should not consume more than 255.5 mg of nitrates daily [14]. According to the available information, the amount of vegetables used in the food basket of Iranian households are 58 g/d of leaf vegetables (leeks, parsley, beet leaves and so on); 7 g/d of peas and beans; 68 g/d of potatoes; and 39 g/d of radishes, garlic and onion bulbs [15]. Therefore, given the continuous and permanent presence of vegetables in people’s food baskets and their importance in supplying the bulk of vitamins, minerals and antioxidants, as well as the very harmful effects of nitrates in food, it is essential to measure nitrates and how various vegetable preparation processes, such as frying, drying, boiling and freezing, impact the nitrate concentration in vegetables. Therefore, this study’s goal is to measure the amount of nitrates in vegetables that are cultivated in Sanandaj city, the effects of cooking and frying processes on nitrate concentration and, finally, the assessment of the health risks of nitrates in vegetables.

## Materials and methods

This cross-sectional study was performed in 2017. Vegetables are classified into three groups—leafy, fruit vegetables, and root vegetables—in terms of growth location and vegetation shape. The statistical population consisted of vegetables beet leaves (*B*. *vulgaris*), parsley (*P*. *crispum*), basil (*O*. *basilicum*), lettuce (*L*. *sativa*), cabbage (*B*. *oleracea*), fenugreek (*T*. *foenum-graecum*), onions (*A*. *cepa*), radishes (*R*. *raphanistrum*), carrots (*D*. *carota*) and tomatoes (*S*. *lycopersicum*) cultivated on Sanandaj vegetable fields. Sanandaj is located on 35° 18′ 52″ N, 46° 59′ 32″ E. The height of this city is 1373.4 meters above sea level. Sanandaj with an average annual precipitation of 2.5 mm and the average temperature of 13.4 °C is classified as semi-arid regions [16]. According to previous studies, the soil texture of Sanandaj farms is loam-clay or sandy-loam, and it contains 6–36% silt, 12–39% clay and 25–82% sand [17]. Most farmers used macro-fertilizer with NPK formula containing 15% nitrogen, 8% phosphorus, 15% potassium, and 1% zinc at a rate of 1–4 kg/m^3^ [18]. Therefore, this type of fertilizer can increase soil and plant nitrate due to its high nitrogen content.

All chemicals used (include potassium hexacyanoferrate (III), zinc acetate dihydrate, sodium tetraborate and acetic acid glacial) were purchased from Sigma Aldrich at 99% purity and were used without any additional process. Whatman quantitative filter paper Grade 41, Membrane Filter (0.22 and 0.45 μm pore size) purchased from Merck Germany. With a standard deviation of 2.1 mg/kg and a precision of 0.2 and a 95% confidence level, 90 samples were selected at nine periods from 10 vegetable farms in autumn and spring (harvesting seasons). From each farm, ten samples (each 1 kg) of all the mentioned vegetables prepared, at first, non-edible parts were removed, and then the vegetables were washed first with tap water followed by distilled water. The vegetables were dried at 60°C using an oven. The time required for drying each type of vegetable depends on their texture. This time took about 12 and 24 hours for the thin-textured vegetables (such as lettuce, cabbage, fenugreek, radish leaves, beet leaves, parsley and basil) and thick-textured vegetables (such as radishes, tomatoes, onion bulb and carrots), respectively. The moisture content of each vegetable was obtained from the difference between fresh and dry weight [19, 20]. An electric mill was used to grind the vegetables. Because the nitrates in the vegetables is sensitive to light and temperature and it decomposes, dried samples were stored in a freezer at a temperature of -18°C to maintain a longer storage time until the tests[20]. To accomplish the frying process, the required amount of soybean oil (Hengameh™), according to the taste of the people in the study area (about 100 ml) was poured into a stainless steel pan. When the oil temperature reached 190°C, the chopped vegetables were fried for 15 minutes. A stainless steel pot was used for the cooking process. Half of the pot was filled with distilled water (400 ml), and the water in the pot was raised to 90°C. The volume of water in all samples was consistent. After 15 minutes, the cooked vegetables passed through a filter and then were ground into powder after dehydrating and drying. All stages of sample preparation and extraction were carried out according to Food Standards 16721–2, and the nitrate measurements were performed using ion chromatography (Compact IC Plus 882 Model, Metrohm, Switzerland). Briefly, 10 g of dried vegetable powder was placed in a volumetric flask then 400 ml of hot water was added and kept in Bain Marie for about 15 minutes. The solution was then cooled to room temperature and filtered using Whatman quantitative filter paper Grade 41. Finally, 40 ml of filtered solution was taken to determine nitrate concentration. Carrez reagents were used to remove the interfering compounds. To prevent damage to the ion chromatography column and reduce the interference effect of the vegetable dye, the final samples were diluted ten times and filtered using syringe filter (0.45 μm and 0.22 μm). Nitrate concentration experiment was repeated three times in each sample and their mean was presented.

For risk assessment, the nitrate concentration in each type of vegetable and the amount of vegetable consumed by each person during the day were suggested as the basis of the calculation. Then, risk potential and risk indicator were determined for the health risk assessment [15]. It should be noted that the amount of consumption of each vegetable in the Iranian food basket is not specified separately and people use different types of vegetables depending on their tastes. Therefore, the health risk assessment for each vegetable was performed separately. In this study, the risk of non-carcinogenic effects was evaluated by calculating the Non-Carcinogenic Hazard Quotient (NHQ). For a single compound, the NHQ is obtained from Eq 1 [21]

$$NHQ=\frac{CDI}{RFD}$$ (1)

where CDI is the chronic daily intake of chemical in mg/kg of body weight per day, and RFD is the chronic reference dose for the contaminant, which is 3.65 mg/kg of body weight per day (Eq 2) [21].

$$CDI=\frac{C\times EF\times ED\times IRF\times (\frac{Kg}{1000g})}{LT\times BW\times (365\frac{day}{year})}$$ (2)

Note that C is the nitrate concentration in vegetable tissue (mg/kg), BW is body weight (for adults of ~70 kg), ED is exposure duration (70 years), EF is exposure frequency (365 days per year), IRF is the average daily intake of vegetables based on the type of vegetable (g/day) and LT refers to the average length of life—70 years for non-carcinogenic effects. The amount of CDI for each kind of vegetable is calculated and, given the specified amount of nitrate RFD, the amount of health risk was obtained for all vegetable types.

To measure soil nitrate levels, sampling from three points at each farm was carried out at depths from 0 to 30 cm, and three samples from each farm were combined. About 1 g of the sample was dissolved in 100 ml of distilled water and filtered using Whatman filter paper No. 42 before being filtered using a Mixed Cellulose Esters Membrane filter. The nitrate amount in the filtered solution was measured using ion chromatography [22]. To measure water nitrate levels, sampling was performed from irrigation water. The water sample was put into polyethylene containers and filtered with Whatman filter paper No. 42, and then nitrate levels were measured using ion chromatography.

### Statistical analysis

SPSS software was used for data analysis. For quantitative variables mean, maximum, minimum and standard deviation and for qualitative data frequencies and percentages were calculated, respectively. To evaluate the normality of the quantitative data, a Kolmogorov–Smirnov test was used before analysis. The significance of the relationship between different parameters was considered with regard to an error rate of 5% and a 95% confidence. A one-way ANOVA test was used to examine the significance relationship between vegetable nitrate levels and vegetable type (leafy, fruit and root vegetables), in which vegetables were classified into 10 groups based on type. The significance of the relationship between the vegetables’ nitrate levels and the sampling period was investigated using t-tests. Based on the sampling period, vegetables were evaluated in two groups. Pearson correlation tests were used to study the significant difference between the nitrate levels of raw and cooked vegetables. A Pearson correlation test was used to study the significant difference between the nitrate levels of raw and fried vegetables.

## Results

In total, 10 species of vegetables, including beets, basil, lettuce, parsley, cabbage, fenugreek, onion bulbs, radishes, carrots and tomatoes, were examined. Of all these types of vegetables, nine samples were taken from nine different farms. Vegetables were divided into three categories, including leafy vegetables (beet leaf, basil, lettuce, parsley, cabbage, fenugreek and onion bulbs), root vegetables (radishes and carrots) and fruit vegetables (tomatoes).

Table 1 shows the moisture content and nitrate levels of vegetables, water and soil in all samples, and Table 2 shows the above parameters based on vegetable categories.

**Table 1:** Vegetable moisture content and the nitrate levels of the vegetables, water and soil in all samples.

Parameter	Number of samples	Minimum	Maximum	Average	Standard deviation
Moisture (%)	90	85	98	91.17	2.76
Vegetable nitrate level (mg/kg)	90	527	15394	4708.91	26.00
Water nitrate level (mg/l)	90	14.33	36.12	26.46	6.89
Soil nitrate level (mg/kg)	90	4.35	9.57	6.93	1.79

**Table 2:** Moisture and vegetable nitrate concentration based on vegetable categories.

vegetable categories	Parameter	Number of samples	Minimum	Maximum	Average	Standard deviation
leafy	Moisture (%)	63	85	97	91	2.67
	Vegetable nitrate level in dry matter (mg/kg)	63	1533	15394	5190	27.56
Root vegetables	Moisture (%)	18	87	94	90	1.88
	Vegetable nitrate level in dry matter (mg/kg)	18	1251	6352	3709	15.87
Fruit vegetables	Moisture (%)	9	92	98.00	95	1.87
	Vegetable nitrate level in dry matter (mg/kg)	9	527	7823	3332	21.81

As shown in Table 2, the highest amount of moisture was found in tomato with a mean of 95%, and the lowest was from root vegetables, with an average of 90%. Also, the highest nitrate level was obtained in leafy vegetables with a mean of 5190 mg/kg in dry vegetables and the lowest in fruit vegetables with a mean of 3332 mg/kg in dry vegetables.

**Fig 1:**
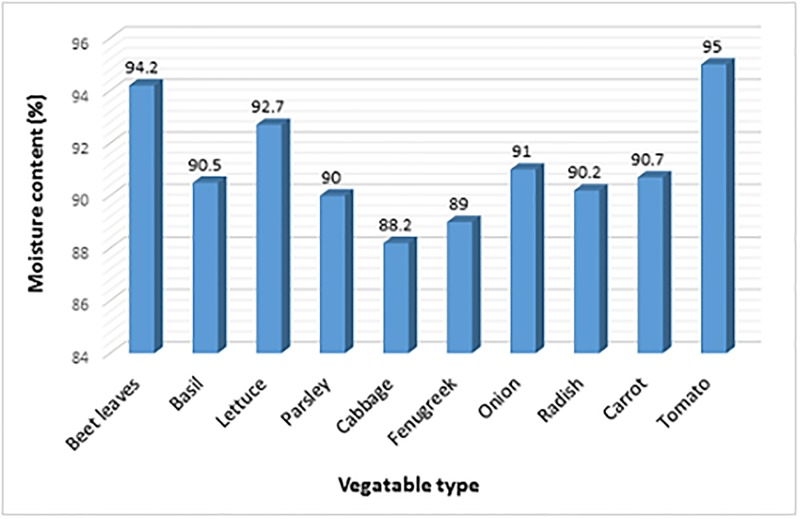
The moisture percentage in each type of vegetable.

According to Fig 1, the highest moisture content was recorded in tomatoes with a mean of 95%, and the lowest was for cabbage with an average of 88.22%.

Based on Table 3, the highest and lowest nitrate levels (ppm) were in beet leaves and tomatoes, with an average of 75.99 ppm and 33.32 ppm, respectively. Results showed that the average nitrate level in leafy vegetables was 5190 mg/kg of dry weight, in root vegetables was 3709 mg/kg of dry weight and in fruit vegetables was 3332 mg/kg of dry weight.

**Table 3:** Nitrate levels of each type of vegetable.

Vegetable categories	Vegetable type	Number of samples	Minimum nitrate (ppm)	Maximum nitrate (ppm)	Nitrate average (ppm)	Nitrate in dry material (mg/kg)	Nitrate in fresh material (mg/kg)
Leafy	Beet leaf	9	31.02	153.94	75.99	7599	439
	Basil	9	29.16	96.85	53.70	5370	507
	Lettuce	9	24.35	88.02	51.36	5136	370
	Parsley	9	21.01	115.24	56.28	5628	562
	Cabbage	9	15.33	108.73	45.04	4504	530
	Fenugreek	9	19.14	71.81	38.42	3842	422
Root vegetables	Onion bulb	9	29.15	58.06	42.54	4254	382
	Radish	9	16.03	63.52	39.02	3902	381
	Carrot	9	12.51	57.42	35.17	3517	324
Fruit vegetable	Tomato	9	5.27	78.23	33.32	3332	166

As shown in Table 4, the nitrate concertation in all vegetables was less than National Iranian Vegetable Nitrate Standard.

**Table 4:** Nitrate concentration in vegetables and comparisons with their levels in different studies and standard values.

Vegetable type	Nitrate levels in fresh material on Sanandaj farms (mg/kg)	Nitrate levels in various literature (mg/kg)	National Iranian Vegetable Nitrate Standard (mg/kg)
Beet leaf	439	93.4 [23], 1026 [24] 1250 [25], 1021 [26]	1000 [27]
Basil	507	184 [26], 2292 [24]	
Lettuce	370	618 [26], 78.4 [23], 1324 [24], 282 [25]	
Parsley	562	945 [26], 985 [24]	
Cabbage	530	326 [26], 74.4 [23], 209 [24, 28]	
Fenugreek	422	640 [25]	
bulb	382	165 [26], 68.5 [23], 164 [24], 127 [28]	
Radish	381	428 [26], 967 [24]	
Carrot	324	104 [26], 69.8 [23], 296 [24]	
Tomato	166	59 [26],68.4 [29], 43 [24], 34.2 [28]	

As shown in Table 5, the vegetable nitrate concertation in the fall, which averaged 6200 mg/kg in dry vegetables, was higher than in the spring, which had an average of 3217 mg/kg.

**Table 5:** Nitrate levels of vegetables in fall and spring.

Nitrate standard levels in dry material (mg/kg)	Season	Number of samples	Minimum	Maximum	Average	Standard
	Fall	45	2414	15394	6200	2646
	Spring	45	527	6847	3217	1456

As shown in Table 6, the relationship between the vegetable nitrate concertation in species and vegetable type and sampling season was significant (p < 0.05).

**Table 6:** Relationship between vegetable nitrate concertation with vegetable categories, vegetable type and sampling season.

	Relationship	Df	F	Sig
Vegetable nitrate levels and vegetable categories	Intergroup	2	3.913	0.024
	Inside the group	87		
	Total	89		
Vegetable nitrate level (ppm) and vegetable type	Intergroup	9	2.524	0.013
	Inside the group	80		
	Total	89		
Vegetable nitrate level (ppm) and sampling season	Intergroup	1	43.899	0.0001
	Inside the group	88		
	Total	89		

As shown in Table 7, the nitrate concertation in cooked vegetables was lower than that in raw vegetables, and the relationship between the cooking process and the nitrate level of vegetables was significant inverted (p < 0.05), showing that cooking the vegetables decreased the nitrate levels.

**Table 7:** Comparison of nitrate content in raw and cooked vegetable samples and the relationship between cooking process and nitrate concertation.

Vegetable name	Consumption type	Minimum nitrate level in dry material (mg/kg)	Maximum nitrate level in dry material (mg/kg)	Average nitrate level in dry material (mg/kg)	Standard deviation	Sig
Beet leaves	Raw	3102	15394	7599.33	40.39	0.001
	Cooked	2541	13393	6698.22	46.47	
Parsley	Raw	2101	11524	5628.89	28.17	0.0001
	Cooked	1764	10385	4947.77	31.68	
Cabbage	Raw	1533	10873	4504.89	29.35	0.0001
	Cooked	1232	9953	3959.33	31.20	
Carrots	Raw	1251	5742	3517.22	14.90	0.001
	Cooked	806	4981	3045.66	18.68	
Tomatoes	Raw	527	7823	3332.78	21.81	0.001
	Cooked	468	9281	3196.33	25.47	

Table 8 demonstrates that the nitrate level in fried vegetables was higher than in raw vegetables, and the relationship between frying vegetables and vegetable nitrate levels was significant (p < 0.05). The frying process significantly increased the nitrate level in vegetables by 12.46% to 29.93%.

**Table 8:** Comparison of nitrate content in raw and fried vegetable samples and the relationship between the vegetable frying process and nitrate levels in vegetables.

Vegetable	Consumption type	Minimum nitrate level in dry material (mg/kg)	Maximum nitrate level in dry material (mg/kg)	Average nitrate level in dry material (mg/kg)	Standard deviation	sig
Beet leaves	Raw	3102	15394	7599.33	40.39	0.001
	Fried	4143	19775	9349.22	49.92	
**bulbs**	Raw	2915	5806	4254.89	9.28	0.0001
	Fried	3623	7320	5388.56	11.20	
Carrots	Raw	1251	5742	3517.22	14.90	0.0001
	Fried	1792	7134	4569.89	17.73	
Tomatoes	Raw	527	7823	3332.78	21.81	0.0001
	Fried	754	5942	3748.38	18.48	

Table 9 reveals that the highest health risk in raw vegetables can be ascribed to parsley at 0.1277, and the lowest is in tomatoes at 0.0465. The highest health risk was in cooked parsley with a level of 0.1248, and the lowest was in tomatoes at 0.0447. Also, the highest health risk in the fried vegetables was in beet leaves at 0.1298, and the lowest was ascribed to tomatoes (0.0524).

**Table 9:** Health risks of nitrates in raw, cooked and fried vegetables based on the food basket of Iranian households for each vegetable.

Vegetable type	Consumption type	The amount of nitrates in the vegetable (mg/kg)	Vegetable consumption (g/day)	Daily chronic absorption of contaminants	Health risk
Beet leaves	Raw	439	58	0.3630	0.0994
	Cooked	410		0.3397	0.0930
	Fried	573		0.4740	0.1298
Basil	Raw	507	58	0.4200	0.1150
Lettuce	Raw	371	58	0.3074	0.0842
Parsley	Raw	563	58	0.4664	0.1277
	Cooked	550		0.4557	0.1248
Cabbage	Raw	531	58	0.4399	0.1205
	Cooked	528		0.4374	0.1198
Fenugreek	Raw	423	58	0.3504	0.0960
**bulbs**	Raw	383	39	0.2133	0.0584
	Fried	532		0.2964	0.0812
Radishes	Raw	382	39	0.2128	0.0583
Carrots	Raw	324	39	0.1805	0.0494
	Cooked	309		0.1721	0.0471
	Fried	464		0.2585	0.0708
Tomatoes	Raw	175	68	0.1700	0.0465
	Cooked	168		0.1632	0.0447
	Fried	197		0.1913	0.0524

As shown in Table 10, the highest health risk when eating 400 g of vegetables per day was among the raw vegetables; this risk was ascribed to parsley at 0.8792. The lowest was tomatoes with a value of 0.2740. In fried vegetables, the highest health risk was in beet leaves at 0.1298, and the lowest was tomatoes (0.0524). The highest level of health risk was ascribed to parsley in raw vegetables with a health risk of 0.1277, and the lowest was seen in tomatoes at 0.0465. The highest health risk in cooked vegetables was parsley, with a level of 0.1248, and the lowest was in tomatoes at 0.0447. In addition, the highest health risk among raw vegetables when eating 400 g of vegetables per day was from parsley at 0.8792, and the lowest was tomatoes with a value of 0.2740. Among the cooked vegetables, the highest risk was from parsley with a level of 0.8610, and the lowest was from tomatoes with a rate of 0.2630. In fried vegetables, the highest risk was from beet leaves (0.8948), and the lowest was tomatoes (0.3076).

**Table 10:** Health risks of nitrates in raw, cooked and fried vegetables in accordance with the FAO/WHO standard (400 g vegetable daily intake).

Vegetable type	Consumption type	Nitrate level in vegetables (mg/kg)	Vegetable consumption (g/day)	Daily chronic absorption of contaminants	Health risk
Beet leaves	raw	439	400	2.5058	0.6872
	cooked	410		2.3428	0.6418
	fried	573		3.2661	0.8948
Basil	raw	507	400	2.8899	0.7917
Lettuce	raw	371	400	2.1147	0.5793
Parsley	raw	563	400	3.2091	0.8792
	cooked	550		3.1428	0.8610
Cabbage	raw	531	400	3.0267	0.8292
	cooked	528		3.0171	0.8266
Fenugreek	raw	423	400	2.4111	0.6605
Onion bulb	raw	383	400	2.1831	0.5981
	fried	532		3.0324	0.8307
Radishes	raw	382	400	2.1774	0.5965
Carrots	raw	324	400	1.8468	0.5059
	cooked	309		1.7657	0.4837
	fried	464		2.6448	0.7246
Tomatoes	raw	175	400	1	0.2740
	cooked	168		0.9600	0.2630
	fried	197		1.1229	0.3076

## Discussion

In this study, we found that the average moisture content in all vegetables was 91.17%. The lowest moisture was 85%, and the highest was 98%. The average nitrate level in all vegetables was 4708.91 mg/kg in dry vegetables, with the lowest and highest nitrate levels in vegetables being 527 mg/kg in dry vegetables and 15394 mg/kg in dry vegetables, respectively. Our findings are consistent with those of Pourmoghim and colleagues, who determined that the average moisture content of all samples was 90%, with the highest moisture being 85.94% and the lowest being 81.34%. Thus, the small differences in moisture levels can be explained by—among other things—weather conditions, irrigation conditions and moisture levels (32). In a study conducted by Shahbazzadegan et al., the highest and lowest concentrations of vegetable nitrates were 35075 and 299 mg/kg of dry weight, respectively, and the reason for this difference is the variety of the examined vegetables, the difference in weather conditions, the soil, the fertilizers used and the cultivation frequency[26].

We also found that the irrigation water nitrate concentration on farms was between 14.31 mg/l and 36.12 mg/l. Moreover, the nitrate levels in the soil were between 4.35 and 9.57 mg/kg, which is less than the critical levels (20 to 22 mg/kg, according to various studies) on all the farms sampled (33). According to a study by Mehrabi et al., the water nitrate levels on farms was 2.53 to 69.7 ppm, and the soil nitrate content ranged from 4 to 33 mg/kg. Another study carried out in the Tehran suburbs showed that soil nitrate concentrations at a depth of 2.5 m were more than 40 mg/kg. A comparison of this study’s results with Mehrabi et al. and other studies showed that the nitrate concentration in the soil is quite variable and mainly relates to the application of fertilizer; however, the type of fertilizer and the rate and time of fertilization can cause changes in the nitrate concentration in the soil. The findings of this study showed that the nitrate concentration in mg/kg of dry weight in different studied vegetables is ordered as follows: leafy > root > fruit. This shows that there is a downward trend in the amount of nitrates in leafy, root and Fruit vegetable, respectively. These findings are consistent with a study conducted by Shahbazzadegan and colleagues, where the nitrate concentration in leafy vegetables was higher than in the root and root vegetables [26]. In a study carried out by Pirsaheb et al., the average nitrate concentration in leafy vegetables was higher than in root vegetables, and it was the lowest in Fruit vegetable [30]. A study conducted by Jafari et al. found that the average nitrate concentration in leafy vegetables was higher than than in root vegetables [31]. Several studies have demonstrated that the nitrate concentration in vegetables is related to different factors, such as the biological properties of the plant, light intensity, soil type, temperature, moisture, plant density/seed, plant maturity, growth period, harvest time, plant unit size, storage time and nitrogen source [23, 32]. Moreover, it has been shown that nitrates are formed in leaf mesophilic cells; fruits and seeds have low nitrate levels, and nitrates are exclusively transported by xylem, which occurs mainly in leaves [33, 34]. It was thus found that the nitrate concentration in vegetables adheres to the following order: leaf > stem > root > inflorescence > gland > fruit > grain [35]. According to Fig 1, the average moisture content of vegetables, such as beet leaves, basil, lettuce, parsley, cabbage, fenugreek, onion bulb, carrots and tomatoes was 94.22%, 90.55%, 92.77%, 90.00%, 88.22%, 89.00%, 91.00%, 90.22%, 90.77% and 95.95% respectively, which is consistent with the amounts reported by Shahbazzadegan et al [26].

We further found that the average nitrate level of beet leaves was 439 mg/kg in fresh vegetables, while the nitrate level of beet leaves varied in other studies and was reported at between 93.4 and 1250 mg/kg [24]. Furthermore, the highest nitrate level in raw vegetables was seen in beet leaves (with an average of 7599 mg/kg), and the lowest nitrate amount was seen in tomatoes (with an average of 3332 mg/kg). However, in a study by Taisser et al., the highest nitrate levels were related to spinach and beet leaves [36]. In this study, the nitrate content seen in all vegetables was lower than the standard level [27]. The nitrate concentration in vegetables is determined via different factors, such as the biological properties of the plant, light intensity, soil type, temperature, moisture, plant density/seed, plant maturity, growth period, harvest time, plant size, storage time and nitrogen source [23, 32]. In addition, we found a significant relationship between vegetable nitrate levels and vegetable categories, vegetable type and sampling season (p < 0.05). Based on studies by Pourmoghim et al., Tabatabaee et al. and Ierna, nitrate levels were higher in leafy, root and Fruit vegetable, respectively [29, 37, 38]. Moreover, the nitrate levels in vegetables in the fall were higher than that in the spring.

This correlation has also been reported by other researchers. For instance, Pourmoghim et al. showed that the nitrate concentration in tomatoes and potatoes in winter was higher than in the summer[37]. Shahlaei et al. indicated a significant relationship between the nitrate levels of vegetables and the sampling season and the type and species of vegetable [28]. Brkić et al. showed that the nitrate levels of vegetables in the fall were higher than in the spring [39]. This difference can be attributed to various factors, such as the duration and intensity of light radiation, soil and weather temperature, moisture and plant age [8, 39–41]. Extreme temperatures reduce the nitrate levels of plants through the assimilation process. Since there are more cloudy days in the fall than in the spring and the air temperature is lower, this results in lower nitrate assimilation during the fall, so the vegetables have higher nitrate levels [42, 43].

We also found that the nitrate levels in cooked vegetables were lower than those in raw vegetables. The cooking process significantly reduced the nitrate levels in vegetables from 4.26 to 15.48%, and the relationship between raw vegetable nitrate content and the amount of cooked vegetables was significant (p < 0.05). Cooking changing the amount of nitrates in vegetables has also been reported by other researchers. Prasad et al. reported that the cooking process reduced the nitrate content of vegetables by 47 to 56% [10]. Sadeghi et al. showed that the cooking process caused a slight increase in nitrate levels of some vegetable categories and a slight decrease in nitrate levels in others [44].

In justifying the role of cooking processes in reducing nitrate levels, it is important to note that nitrates have a high tendency to dissolve in water, and when vegetables are immersed in water, the nitrates tend to move along the diffusion gradient from being more concentrated (inside the vegetable) to being less concentrated (inside the water in which the vegetable is located). Increasing temperature and time will facilitate the diffusion process and the movement of nitrates from the inside the vegetable into the distilled water, and finally, more nitrates will be released from the vegetable into the water, which will reduce the nitrate levels in the vegetable [10, 45].

Our study also indicates that the nitrate level in fried vegetables was higher than in raw vegetables. The frying process significantly increased the nitrate levels in vegetables. Our findings are consistent with other studies that found that the frying process increases the nitrate level of vegetables [1, 10, 46]. Studies have shown that increasing the concentration of vegetable nitrates after frying can be related to the reduction of the vegetable’s mass, which ultimately results in the condensation of nitrates in the sample and the amount of oil consumed. This can be explained by the fact that during the frying process, vegetable nitrates remain at the same level, but the volume or weight of the vegetable is reduced; this causes the fixed amount of nitrates to be crowded into a lower volume. Nevertheless, the type of oil used for frying may be effective in reducing the amount of nitrates in the vegetable; that is, the oil itself may contain nitrates. This hypothesis has been well documented in that the roots of immature plants, such as soybeans, have specific bacteria that stabilize elemental nitrogen to ammonia in plants. The ammonia is converted into nitrates through the nitrification process and by other microorganisms in soy plants, and because soybean oil was used in this study, the oil itself could contain nitrates and may have contaminated the fried vegetables [10, 47].

The average amount of any kind of vegetable in Iran’s food basket is clear. Accordingly, 58 g/day of leafy vegetables (leeks, parsley, beet leaves, etc.); 7 g/day of peas and beans; 68 g/day of potatoes and tomatoes; and 39 g/day of radishes, garlic and bulbs are consumed from the food basket of Iranian households [15]. According to the WHO, the average vegetable consumption per person is 400 g/day [24]. Therefore, the health risk for each individual vegetable is calculated based on the amount of consumption in the food basket of Iranian households and based on the 400 g/day intake. In this study, the highest health risks regarding nitrates in vegetables are fried vegetables, raw vegetables and cooked vegetables, respectively. Nitrate risks from consuming vegetables is high regarding the consumption of 400 g/day and, in some cases, is very close to the maximum permissible health risk. The highest health risk was related to fried beet leaves at 0.8948, and the least risk was from raw tomatoes with a level of 0.2607. In other studies, such transparency on the health risks of nitrates in vegetables has not been discussed. In a study conducted in Korea in 2013, the health risk was estimated for 400 g/day. According to the study, the daily intake of nitrates per unit of body weight for each of the vegetables was less than 1. Due to the low daily intake of nitrates compared to the maximum permissible daily intake, the health risk in all cases was lower than 1, meaning that the health risk was lower than the standard limit. In this study, the highest and lowest health risks were related for spinach and bean sprouts with a daily intake of 0.235 and 0.00 mg/kg/day, respectively. Many of the vegetables used in this research were not studied, but considering that the highest health risk was related to vegetables with the highest nitrate concentrations, the lowest health risk was related to vegetables with the lowest nitrate concentrations; therefore, the health risk is directly related to the nitrate concentrations in vegetables, and the results of that study are consistent with this study [48].

In another study by Gruszecka et al., the acceptable daily intake of nitrate (ADI) was considered to be 3.7 mg/kg body weight and the NHQ formula was used to assess health risk. The health risk of consuming several vegetables, including carrots and tomatoes, was far below the permitted level (HQ<1). Also, the health risk for some other vegetables, such as beets and cabbage, was higher than carrots and tomatoes, but the health risk for these vegetables was also below the standard (HQ<1), and in some cases the health risk of vegetables consumption was close to the limit of acceptable risk value [49]. Therefore, the results of this research are similar to those of the present study. It should be emphasized that consuming vegetables is only one way to intake nitrate. Thus a health risk of less than 1 in this condition cannot alone indicate a healthy level of nitrate intake. Therefore, other sources of nitrate such as drinking water and other foodstuff should be considered to determine the health risk of nitrate.

## Conclusion

In this study, nitrate concentration in vegetables, the effect of home cooking processes on nitrate reduction and assessment of health risks of nitrate in vegetables for consumers were investigated. The obtained research results indicate that, nitrate concentrations were higher in tomato and onion bulb but lower in other vegetables. Nitrate concentration in leafy vegetables was higher than root and fruit vegetables and these values were higher in autumn than in spring. The results of this study indicate that the cooking process reduces vegetable nitrate levels and lowers the health risk of eating raw vegetables, while the frying process increases the nitrate level in vegetables and thus increases their health risk. The highest health risk level in raw, cooked and fried vegetables was related to parsley, parsley and beet leaves, respectively, and the lowest in all categories was tomatoes. Generally, the highest health risk was related to fried beet leaves, and the lowest was raw tomatoes. Therefore, it is necessary to consume fewer fried vegetables, and most vegetables should be consumed raw. Also, based on this study, the consumption of cooked vegetables is not problematic. In most cases, the nitrate levels in raw vegetables in Sanandaj is acceptable (lower than the standard). Considering the high benefits of vegetables, their consumption in terms of nitrates is unlikely to be problematic.
